# Lipidomic Impacts of an Obesogenic Diet Upon Lewis Lung Carcinoma in Mice

**DOI:** 10.3389/fonc.2018.00134

**Published:** 2018-05-11

**Authors:** Sneha Sundaram, Petr Žáček, Michael R. Bukowski, Aaron A. Mehus, Lin Yan, Matthew J. Picklo

**Affiliations:** ^1^USDA-ARS Grand Forks Human Nutrition Research Center, Grand Forks, ND, United States; ^2^Department of Chemistry, University of North Dakota, Grand Forks, ND, United States

**Keywords:** lipidomics, Lewis lung carcinoma, primary tumor, high-fat diet, mice

## Abstract

Metabolic reprogramming of lipid metabolism is a hallmark of cancer. Consumption of a high-fat obesogenic diet enhances spontaneous metastasis using a Lewis lung carcinoma (LLC) model. In order to gain further insights into the mechanisms by which dietary fats impact cancer progression, we conducted a lipidomic analysis of primary tumors originated from LLC from mice fed with a standard AIN93G diet or a soybean oil-based high-fat diet (HFD). Hierarchical clustering heatmap analysis of phosphatidylcholine (PC) lipids and phosphatidylethanolamine (PE) lipids demonstrated an increase in polyunsaturated fatty acids (PUFA)-containing phospholipids and a decrease in monounsaturated fatty acids (MUFA)-containing lipids in tumors from mice fed the HFD. The quantities of 51 PC and 24 PE lipids differed in primary tumors of LLC from mice fed the control diet and the HFD. Analysis of triacylglycerol (TAG) lipids identified differences in 32 TAG (by brutto structure) between the two groups; TAG analysis by neutral loss identified 46 PUFA-containing TAG species that were higher in mice fed with the HFD than in the controls. Intake of the HFD did not alter the expression of the *de novo* lipogenesis enzymes (fatty acid synthase, acetyl-CoA carboxylase-1, and stearoyl-CoA desaturase-1). Our results demonstrate that the dietary fatty acid composition of the HFD is reflected in the higher order lipidomic composition of primary tumors. Subsequent studies are needed to investigate how these lipidomic changes may be used for targeted dietary intervention to reduce tumor growth and malignant progression.

## Introduction

Obesity, resulting from an imbalance between energy intake and energy expenditure, is a leading risk factor for cancer. Excess body fat mass is positively associated with the risk of multiple cancers at different organ sites (1, 2). Furthermore, cancer patients who are overweight or obese are at a greater risk of recurrence and shorter disease-free intervals compared to patients with normal body weight (3–5). In experimental animals, obesity enhances both primary tumorigenesis (6) and metastasis (7, 8). Multiple studies demonstrate that obesity and type of dietary lipid intake modify lipidomic profiles in humans (9, 10) and laboratory rodents (11, 12). Understanding how the tumorigenic lipid profile responds to obesity and dietary lipid intake may provide insights into novel preventive and therapeutic strategies.

Clinical studies show altered lipid metabolism in cancer patients (13–15). Phosphatidylcholines (PC), phosphatidylethanolamines (PE), and sphingomyelins are the most dominant phospholipids (PL) comprising 80% of the cell membranes (16). Patients with breast (17), lung (18), and ovarian cancer (19) exhibit elevated concentrations of PCs and PEs in cancerous tissues compared to adjacent normal tissues. On the other hand, significantly lower concentrations of total PEs in plasma are related to clinical stage and pathologic grade of prostate cancer (20). The levels of ceramides (CER), a dominant member of the sphingomyelin family, are elevated in head and neck squamous cell carcinoma compared to the normal tissue (21). The expression of ceramide synthase mRNAs is higher in cancerous breast tissue than in normal breast tissue (22). Triacylglycerols (TAG), in the form of lipid droplets, are the main constituents of body fat. Increased numbers of lipid droplets in colon adenocarcinoma has been suggested to be active in regulation of cancer pathogenesis (23). It is proposed that these altered cellular lipid profiles and distribution change cellular functions, which may affect the development and progression of cancer (24).

Lewis lung carcinoma (LLC) is a highly malignant carcinoma commonly used in murine studies of metastasis (7, 8), angiogenesis (25), and cachexia (26). When LLC cells are subcutaneously transplanted, they produce a rapidly growing primary tumor that metastasizes to the lungs. We found that obesity enhances the aggressiveness of pulmonary metastasis of LLC (7, 8). The purpose of this study was to determine changes in lipidomic signatures in primary tumors of LLC from mice caused by the intake of a high-fat, obesogenic diet.

## Materials and Methods

### Chemicals

Chloroform, potassium chloride, butylated hydroxytoluene (BHT), ammonium chloride, and ammonium acetate were purchased from Sigma-Aldrich (St. Louis, MO, USA). Methanol was from Avantor Performance Materials, Inc. (Center Valley, PA, USA). PL and CER standards, including LIPID MAPS mass spectrometry (MS) standards—Core H, were from Avanti Polar Lipids Co. (Alabaster, AL, USA). The compounds PC(17:0/14:1), PC(17:0/20:4), PC(21:0/22:6), LPC(17:1), PE(17:0/14:1), and PE(21:0/22:6) of certified concentrations were used as the internal standards for quantitation of PCs, lyso PCs (LPCs), CERs, PEs, and lyso PEs (LPEs). TAG species were from Nu-Chek Inc. (Elysian, MN, USA). Antibodies against fatty acid synthase (FASN), acetyl-CoA carboxylase-1 (ACC1), stearoyl-CoA desaturase-1 (SCD1) were from Cell Signaling Technology (Danvers, MA, USA), that against phosphorylated ACC1 (pACC) was from EMD Millipore (Burlington, MA, USA), and that against the system control protein was from ProteinSimple (San Jose, CA, USA).

### Animals and Diets

Three-week-old male C57BL/6 mice (Harlan, Madison, WI, USA) were maintained in a pathogen-free room with a temperature of 22 ± 1°C and a 12:12 h light/dark cycle. The standard AIN93G diet (27) providing 16% of energy from soybean oil and a modified AIN93G diet providing 45% of energy from soybean oil [high-fat diet (HFD)] were used in this study (Table 1). Both diets were powder diets and were stored at −20°C until feeding.

**Table 1:** Composition of experimental diets.

	AIN93G	High-fat
Ingredient	g/kg	g/kg
Corn starch	397.5	42.5
Casein	200	239.4
Dextrin	132	239.4
Sucrose	100	119.7
Soybean oil	70	239.4
Cellulose	50	59.8
AIN93 mineral mix	35	41.9
AIN93 vitamin mix	10	12
l-cystine	3	3.6
Choline bitartrate	2.5	3
*t*-Butylhydroquinone	0.014	0.017
Total	1,000	1,000
Energy	%	%
Protein	20	20
Fat	16	45
Carbohydrate	64	35
Analyzed gross energy kcal/g^a^	4.3 ± 0.1	5.2 ± 0.1

*^a^Values are mean ± SD of three samples analyzed from each diet*.

### Lewis Lung Carcinoma

The LLC cell line, a variant that metastasizes to lungs (28), was obtained from Dr. Pnina Brodt, McGill University, Montreal, Quebec, Canada. The cells were cultured with RPMI-1640 medium containing 10% heat-inactivated fetal bovine serum and maintained in a humidified atmosphere of 5% CO_2_ in air at 37°C. Hoechst DNA staining and direct culture tests (performed by American Type Cell Collection, Manassas, VA, USA) showed that cells were free of mycoplasma.

### Experimental Design

After acclimation with the AIN93G diet for 1 week, mice were randomly assigned into two groups (*n* = 6 per group) and fed the AIN93G diet or the HFD. Mice had free access to their diets and deionized water. Ten weeks after initiation of the experimental feeding, mice were subcutaneously injected with 2.5 × 10^5^ viable LLC cells per mouse into the lower dorsal region. Intake of the HFD for 10 weeks was sufficient to demonstrate an elevation in adiposity as observed in our previous work (29). Mice were assessed for body composition 1 week before cell injection by using an Echo Whole-Body Composition Analyzer (Model 100, Echo Medical System, Houston, TX, USA). Ten days after cell injection when the resulting subcutaneous tumor was approximately 1 cm in diameter, mice were anesthetized with a mixture of ketamine and xylazine; the subcutaneous tumors were harvested and cleaned to remove skin, weighed, flash-frozen in liquid nitrogen, and stored at −80°C.

### Lipidomic Analysis

The primary tumor (approximately 100 mg) was homogenized with 2 mL of extraction solution (hexane:isopropanol 1:1, 50 µM BHT) and centrifuged at 2,000 *g* for 10 min at 10°C. The liquid phase was collected. The process was repeated once. The pooled liquid phases were evaporated under an N_2_ stream at 30°C. The dried extract was reconstituted in 1 mL of chloroform:methanol (1:1) to yield a primary extract. The extract (10 μL) was mixed with 980 μL of mobile phase (chloroform: methanol 1:1, 10 mM ammonium acetate, 50 μM of BHT) and 10 μL of a CER(d18:1/17:0) to yield a secondary extract for MS analysis. This analysis determined isobaric and isomeric PCs using MS^3^ fragmentation (LPC fragments were employed), isobaric PEs using MS^2^ scan (fatty acid fragments were employed), and CER analysis using precursor ion scan (PIS) *m/z* +264. The secondary extract (25 µL) was further mixed with 975 µL of mobile phase and spiked with 15 µL of a mixture of PC and LPC internal standard and 8 µL of PE internal standard for analysis of PC, LPC, PE, and LPE using experiments PIS *m/z* +184 and neutral loss scan (NLS) *m/z* +141, respectively. The mass spectrometric analysis for PCs and PEs were performed using the methods previously described (30, 31). In addition to the positive mode, PEs were analyzed using the MS^2^ fragmentation of precursor [M-H]^−^ performed in negative mode (32); this combination enabled identification and quantitation of isobaric PE. Furthermore, analysis of the isobaric PE employing MS^2^ was corrected for possible interferences of PC species. LPEs were quantified only relatively with respect to the PE(17:0/14:1) due to the lack of commercially available LPE internal standards. The identified lipids were normalized by the fresh tumor weight (nmol/g tissue). PL nomenclature was used according to Liebisch et al. (33).

Ceramides were analyzed by using the method of PIS of *m/z* 264 in positive mode (PIS *m/z* +264) (34). The scanned fragment *m/z* 264 resulted from the loss of N-linked fatty acids and two molecules of water. The nature of this fragment that contained the sphingosine backbone enabled direct identification of the amide-linked fatty acids from the mass of the molecular ion [M + H]^+^ or [M + H–H_2_O]^+^. For the quantification purposes only the [M + H]^+^ ion was employed. Parameters for the experiment were set as follows: collision energy 33 V, ion spray voltage 5,200 V, declustering potential 80 V, entrance potential 10 V, and scanning rate 200 Da/s. CER(17:0) was used for quantification of CER. The following CER including the CER(17:0) were employed for an ionization efficiency correction: CER(10:0), CER(20:0), and CER(24:1). Data collection and processing was performed as previously described (31). Along with CER species, PIS *m/z* +264 detected hexosylceramides (HexCer), which were identified by precursor mass. These were quantified following the same method as other CER, resulting in semi-quantitative data that allows for detection of differences between treatment groups.

### Triacylglycerol Analyses

Mass spectrometry was performed for analysis of TAG as previously described (31). The ion source was calibrated with an equimolar TAG standard as previously shown (35). Neutral mass losses were used to determine the relative content of selected fatty acids across brutto structures in multiplexed experiments, and correction factors for other brutto structures were imputed as detailed previously (31).

Triacylglycerol appeared in the mass spectrum as ammonium complexes between *m/z* 750 and *m/z* 1,000, with cluster of TAG species around *m/z* 825, 850, 875, 900, 925, and 950, corresponding to TAG species with fatty acyl chains containing 48, 50, 52, 54, 56, and 58 carbons, respectively. Within these clusters, signals for the monoisotopic species differed in *m/z* by two units, representing the desaturation level across the three acyl chains.

A numerical expression for the distribution of saturation within a given TAG group with C carbons as the summation of the product of the saturation number (*N*) and the mole fraction of the species with that saturation value for saturation values within the group. The resultant weighted desaturation index (*D_C_*) describes the average desaturation level of the group, and is higher for more desaturated species.

$$D_{C}=\sum_{i=0}^{N_{\text{max}}}N_{i}\frac{c_{N_{i}}}{\left(c_{N_{0}}+c_{N_{1}}+\ldots c_{N_{\text{max}}}\right)}$$

### Analysis of *De Novo* Lipogenesis Enzymes

The primary tumor (approximately 35 mg) was pulverized and lysed for protein extraction as previously described (36). Protein concentration was determined by using the Bradford protein assay (Bio-Rad Laboratories, Inc., Hercules, CA, USA).

Diluted protein lysates were combined with Simple Western™ sample dilution buffer (ProteinSimple, San Jose, CA, USA) which contains reducing agent dithiothreitol, fluorescent standards, and a system control protein [26 or 90 kDa-dependent on the molecular weight (MW) kit used]. A ratio of 4:1 (protein lysate-to-sample dilution buffer) was used. The samples were then analyzed on the Sally Sue instrument (ProteinSimple) as previously reported (37). The ProteinSimple system control protein served as an internal control and was also used to normalize protein expression. For the lower MW SCD1, the 26 kDa system control protein was used for normalization and for the higher MW proteins (FASN, ACC1, and pACC), the 90 kDa system control protein was used.

### Statistical Analyses

Resulting lipidomic data were analyzed by using MetaboAnalyst software (version 3.0, McGill University, Sainte Anne de Bellevue, QC, Canada) (38, 39). A false discovery rate (FDR) of 0.05 was used and the FDR-corrected *p*-values are reported (39). Student’s *t*-test was performed to compare differences in weight gain and body composition between the two groups by using SAS software (version 9.4, SAS Institute, Cary, NC, USA). Data are presented as mean ± SD. Differences with a *p* ≤ 0.05 were considered significant.

## Results

Morphometric results are presented in Table 2. Mice on the HFD weighed more than those on the AIN93G control diet. Weight gain was 35% higher in mice fed the HFD than in those fed the control diet (Table 2). The percent body fat mass was 44% higher in mice fed the HFD, but there was no difference in absolute lean mass weight between the two groups (Table 2).

**Table 2:** Weight gain and body composition of mice fed the AIN93G or the high-fat diet.

	AIN93G	High-fat
Weight gain, g	11.6 ± 3.6^b^	15.6 ± 2.1^a^
Fat mass, %	21.3 ± 6.5^b^	30.7 ± 6.2^a^
Lean mass, %	70.5 ± 6.1^a^	62.3 ± 5.7^b^
Lean mass weight, g	21.5 ± 1.2	21.0 ± 0.9

*Values (mean ± SD) with different superscripts are different at *p* ≤ 0.05 (*n* = 6 per group)*.

### Phospholipids

Analysis of PLs identified 169 PC lipids (discriminating isobaric and regioisomeric PC, LPC, PC ethers, and sphingomyelins) and 81 PE lipids (discriminating isobaric species). Values and statistical comparisons for all PC and PE lipids analyzed are provided in Tables S1 and S2 in Supplementary Material.

**Figure 1:**
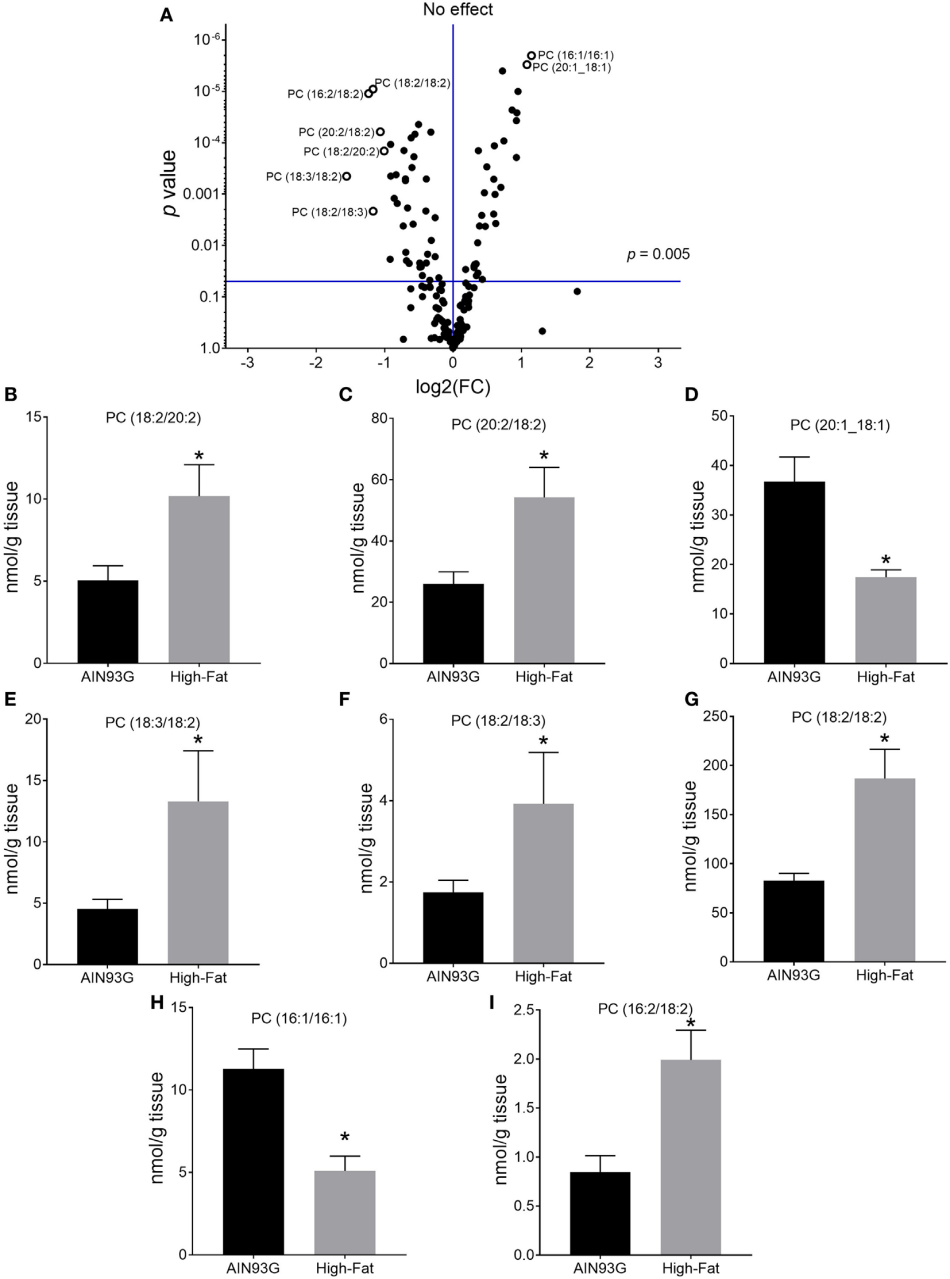
Volcano plot **(A)** and concentrations of selected phosphatidylcholines [PCs; **(B–I)**] in the primary tumor of Lewis lung carcinoma with a twofold or greater difference between the AIN93G diet and the high-fat diet. Values are mean ± SD (*n* = 6 per group). *p ≤ 0.05 for each comparison.

Of the 169 PC species identified, 51 were significant in quantity between the two groups (29 were higher and 22 were lower in mice fed the HFD). A volcano plot (a combination of fold change and *p*-values of comparisons) of PC lipids showed that the difference of eight of the identified PC were at least twofold (Figure 1A); six [PC(18:2/20:2), PC(20:2/18:2), PC(18:3/18:2), PC(18:2/18:3), PC(18:2/18:2), and PC(16:2/18:2)] showed an increase (Figures 1B,C,E,F,G,I) and two [PC(20:1_18:1) and PC(16:1/16:1)] showed a decrease (Figures 1D,H) by HFD. The identity of the 16:2 fatty acid detected in PC(16:2/18:2) was confirmed by GC-MS using authentic standards as the n6 species, 7,10-hexadecadienoic acid.

**Figure 2:**
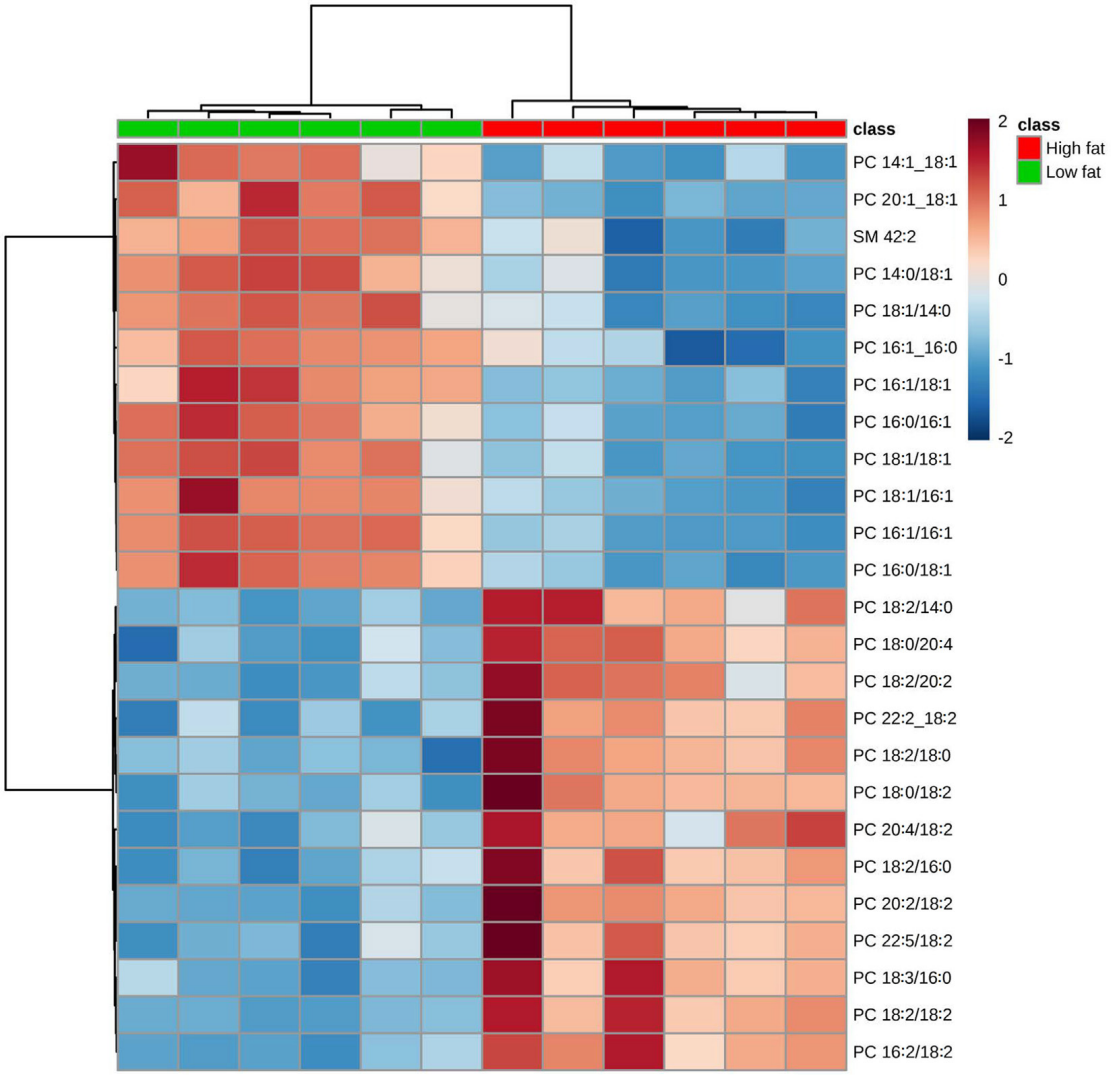
Hierarchical clustering heatmap analysis of phosphatidylcholine (PC) lipids in the primary tumor of Lewis lung carcinoma from mice fed the AIN93G diet or the high-fat diet. Each colored cell on the map corresponds to a concentration value. The top 25 lipid species from the two diet groups are presented and ranked by *t*-tests to retain the most contrasting patterns. Values are measured by Euclidean distance with a Ward clustering algorithm (*n* = 6 per group). *p ≤ 0.05 for each comparison.

Hierarchical clustering heatmap analysis makes the identified lipids visualized and shows the variation of each lipid between the comparisons. The heatmap of PC (Figure 2) showed greater amounts of polyunsaturated fatty acids (PUFA) in tumors from mice fed the HFD than in those fed the control diet, whereas greater amounts of monounsaturated fatty acids (MUFA) were in mice fed the control diet than in those fed the HFD.

**Figure 3:**
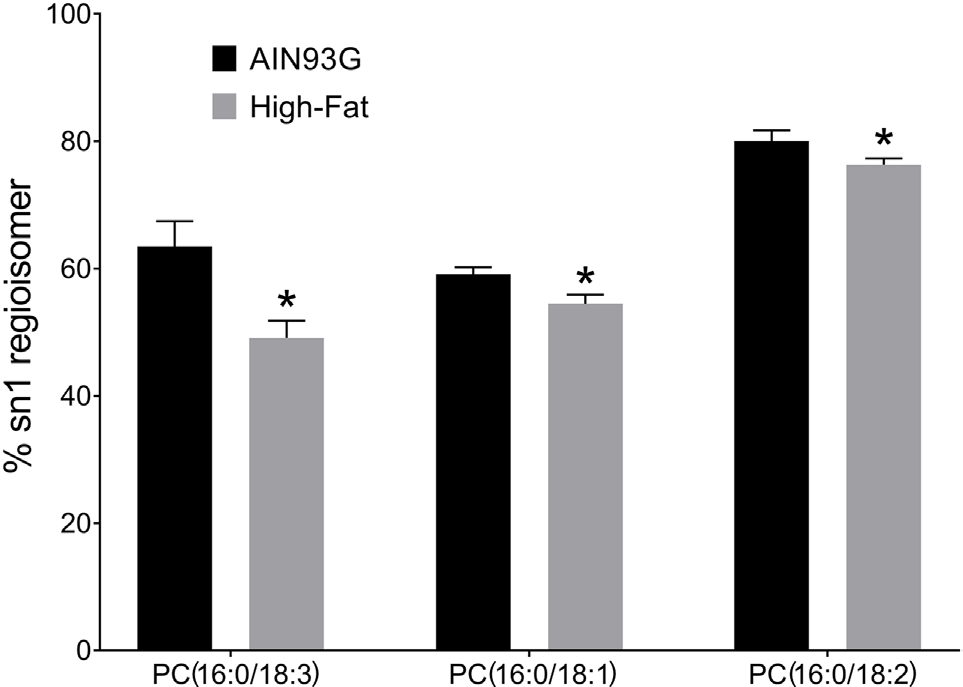
Changes in phosphatidylcholine (PC) regioisomers in primary tumors of Lewis lung carcinoma in response to consumption of high-fat diet. Data are presented as the % of sn1 isomer for each lipid (*n* = 6 per group). *p ≤ 0.05 for each comparison.

We tested the hypothesis that intake of the HFD changed the regioisomeric (sn1 vs sn2) distribution of PC lipids in the tumors. Of the 47 regioisomers analyzed, the percent of the regioisomer in the sn1 vs sn2 form was lower in HFD vs the control for PC(16:0/18:3) (63.5 ± 3.9 vs 49.1 ± 2.7%), PC(16:0/18:2) (80.0 ± 1.7 vs 76.3 ± 1.0%), and PC(16:0/18:1) (59.1 ± 1.1 vs 54.5 ± 1.4%) (Figure 3). These results indicate that for these three PC species, the unsaturated fatty acid was observed in the sn1 position to a greater extent in tumors from mice fed the HFD.

**Figure 4:**
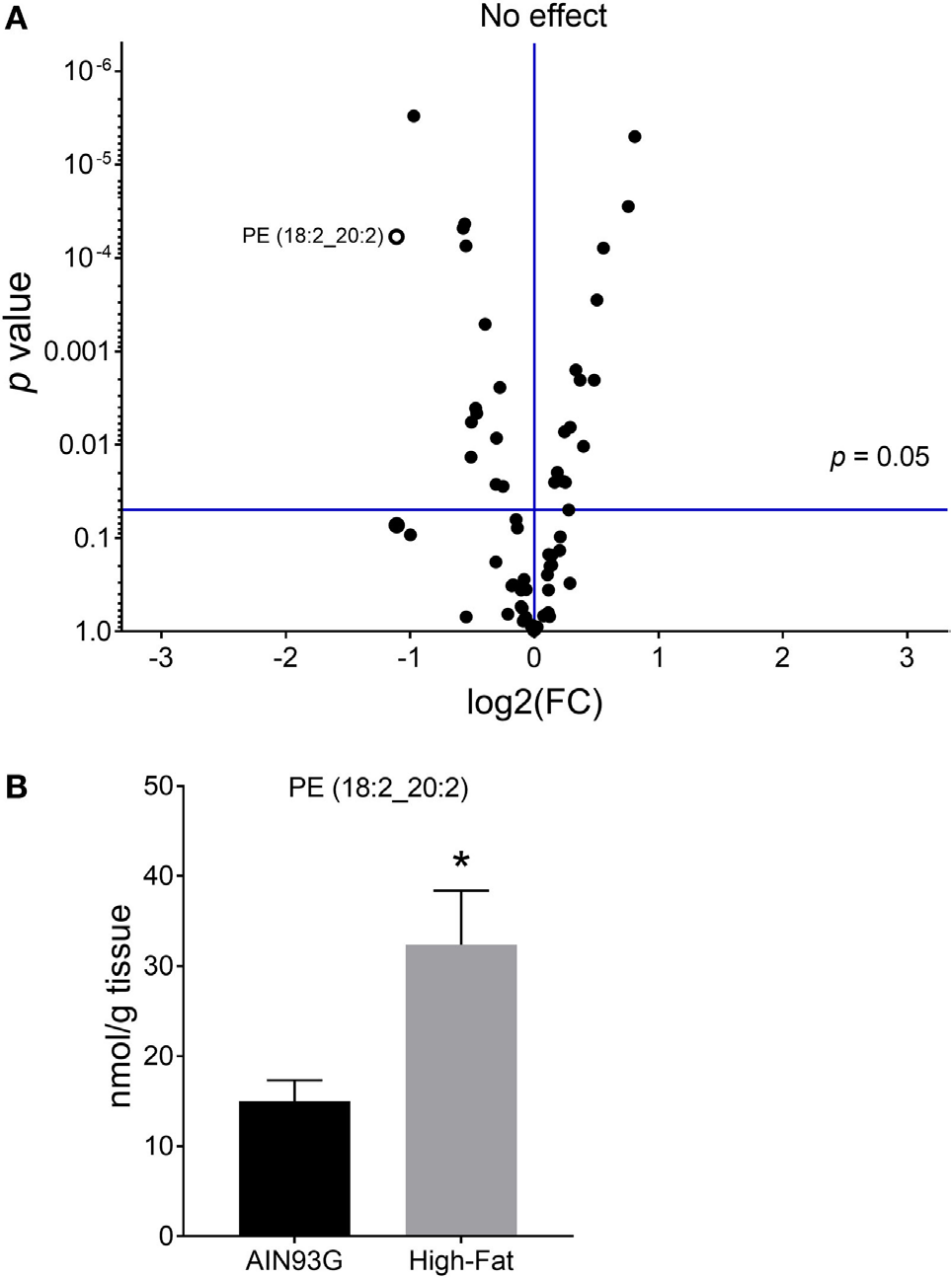
Volcano plot **(A)** and concentration of phosphatidylethanolamine [(PE); 18:2_20:2] **(B)** in the primary tumors of Lewis lung carcinoma with greater than twofold difference between the AIN93G diet and the high-fat diet. Values are mean ± SD (*n* = 6 per group). *p < 0.05 for each comparison.

**Figure 5:**
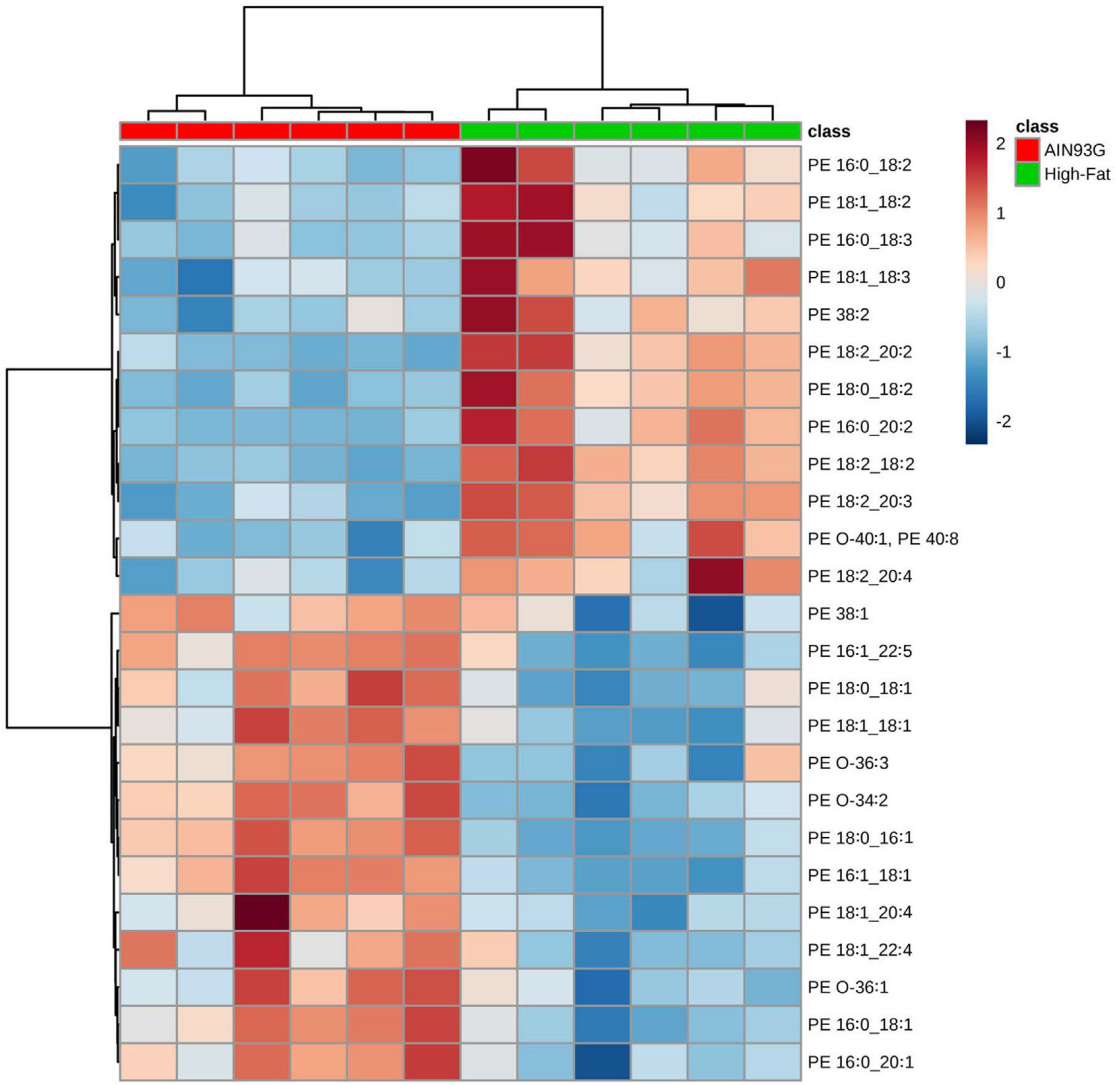
Hierarchical clustering heatmap analysis of phosphatidylethanolamine (PE) lipids in the primary tumor of Lewis lung carcinoma from mice fed the AIN93G diet or high-fat diet. Each colored cell on the map corresponds to a concentration value. The top 25 lipid species from the two diet groups are presented and ranked by *t*-tests to retain the most contrasting patterns. Values are measured by Euclidean distance with a Ward clustering algorithm (*n* = 6 per group). *p ≤ 0.05 for each comparison.

Twenty-four PE are significantly different in quantity between the two groups [12 were higher and 12 were lower in mice fed the HFD (Table S2 in Supplementary Material)]; however, unlike for PC, regioisomers (i.e., sn1 vs sn2) for PEs could not be determined. The volcano plot of PE identified difference in PE(18:2_20:2) (Figure 4A); the amount of PE(18:2_20:2) was twofold greater in mice fed the HFD than in the control mice (Figure 4B). Similar to the PC lipids, there was a shift to an elevation in PUFA-containing PE lipids in tumors from mice fed the HFD as demonstrated by heatmapping with cluster analysis (Figure 5).

### Ceramides and HexCer

Changes in CER and HexCer metabolism have been studied with respect to cancer development (21, 22). Our analyses identified and quantified 15 separate CER species and 5 HexCer species. However, there were no significant differences in CER and HexCer species between the two dietary groups (data not shown). Values and statistical comparisons for CER and HexCer analyzed are provided in Table S3 in Supplementary Material.

### Triacylglycerol

**Figure 6:**
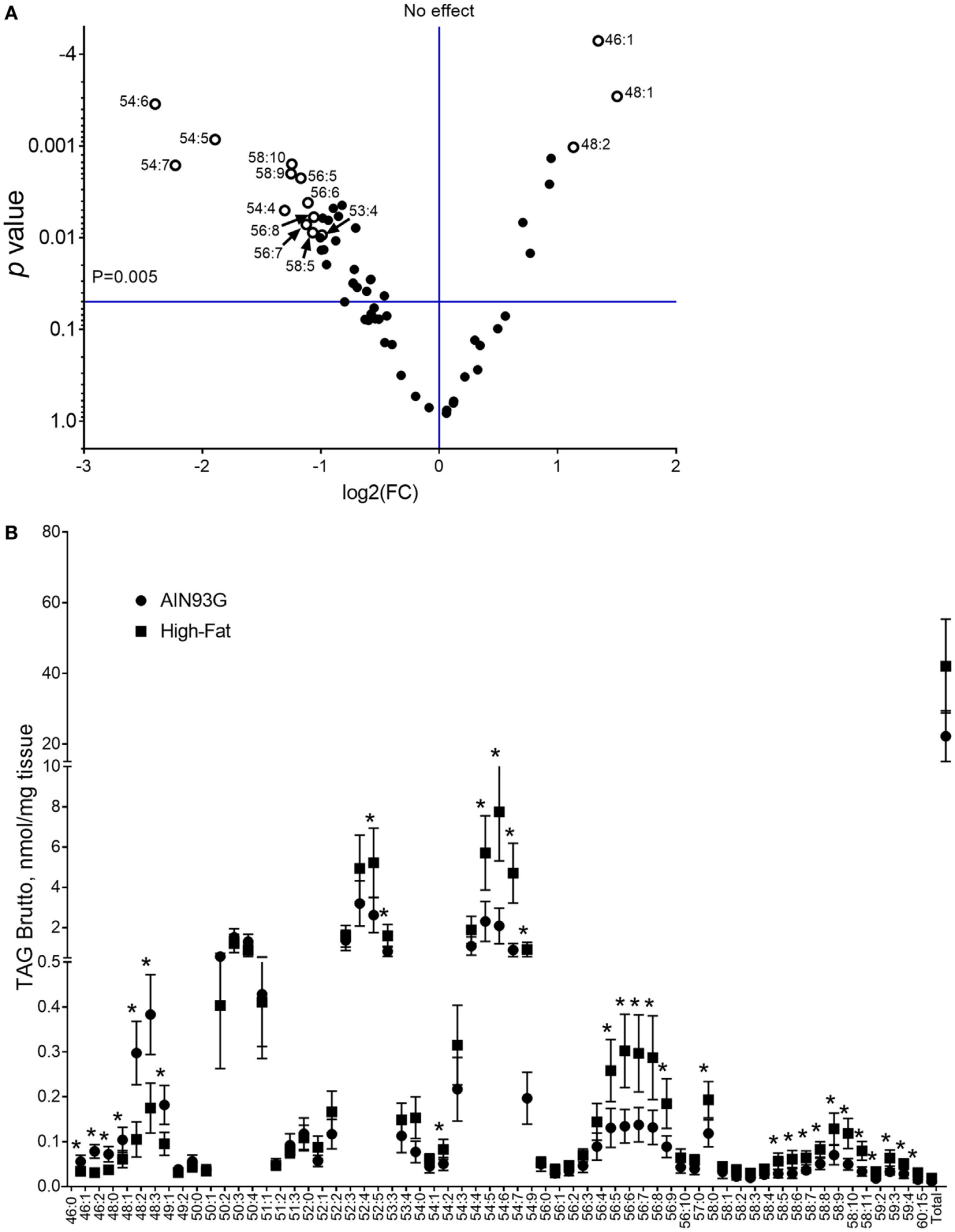
Volcano plot with a twofold difference **(A)** and concentrations of triacylglycerols [(TAG); **(B)**] in the primary tumor of Lewis lung carcinoma from mice fed the AIN93G diet or the high-fat diet (HFD). Values are mean ± SD (*n* = 5 for the AIN93G group and *n* = 6 for the HFD group). *p ≤ 0.05 for each comparison.

Analysis of TAG composition identified 62 separated TAG by brutto structure. The volcano plot analysis of TAGs showed that the difference of 15 of the identified TAGs were at least twofold (Figure 6A). Twelve TAGs (53:4, 54:4, 54:5, 54:6, 54:7, 56:5, 56:6, 56:7, 56:8, 58:5, 58:9, and 58:10) showed significant increases and three (46:1, 48:1, and 48:2) showed significant decreases by the HFD (Figure 6B). Of the 62 TAG structures identified, 32 TAG differed between treatment groups and were significant in quantity between the two diets. Values and statistical comparisons for the TAG concentration (for brutto structure) are provided in Table S4 in Supplementary Material. One of the samples from the control, AIN93G group was omitted from statistical analysis for TAG as its values were 2.5-fold greater than that of the others in the dietary group. Analysis of this sample was repeated with similar results.

**Figure 7:**
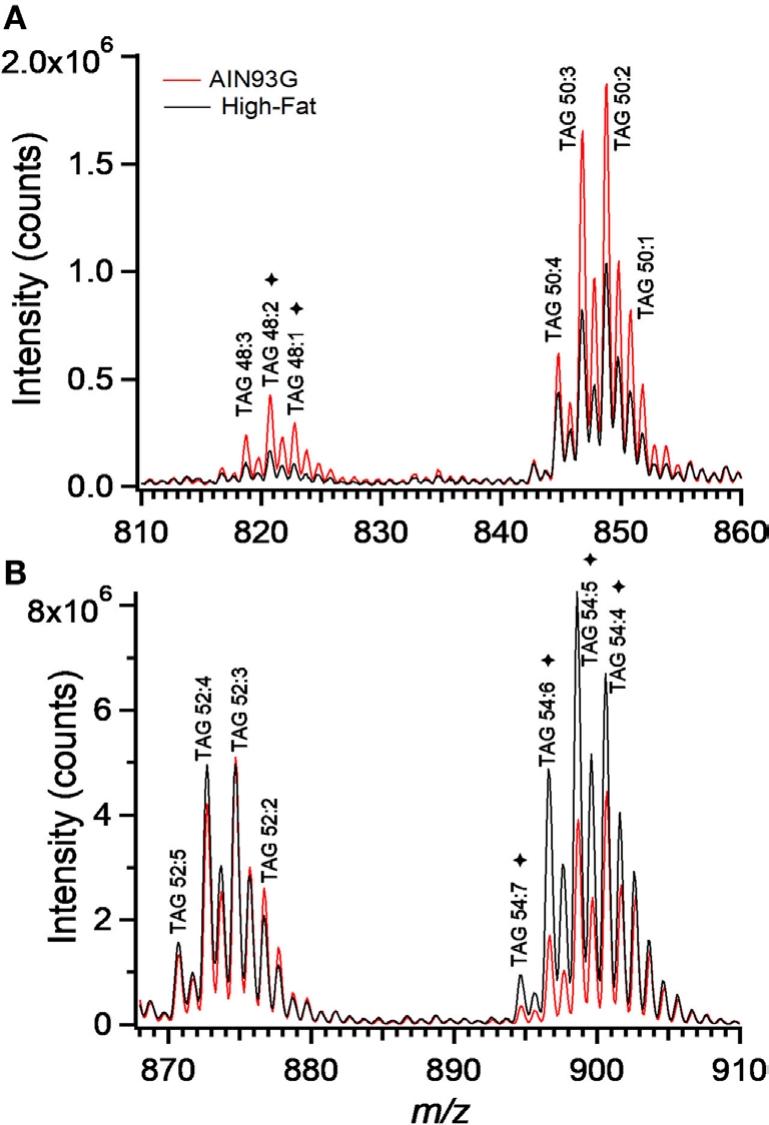
Enhanced mass spectrum for triacylglycerols (TAG) 48 and TAG50 **(A)** and TAG52 and TAG54 **(B)**. Note the higher concentrations of C48 and polyunsaturated C54 containing TAG species in tumors from the AIN93G **(A)** and high-fat diet **(B)** fed mice, respectively. *denote significant differences in TAG species as determined by volcano plot analysis (Figure 6).

Enhanced mass spectra for the four most intense TAG clusters (TAG48, TAG50, TAG52, and TAG54) are shown for the control and the HFD groups. The intensity of signals for TAG48 and TAG50 decreased in the HFD group with TAG48:1 and 48:2 having most significant contribution to this decrease (Figure 7A). Signals for TAG52 remained unchanged; however, TAG54 increased in the HFD group, with TAG54:4, 54:5, 54:6, and 54:7 providing significant contributions (Figure 7B). These results can be expressed in terms of the weighted saturation index (*D_C_*). For the TAG46, TAG48, and TAG50, *D_C_* was unchanged between the control and HFD groups (Table 3). For TAG species with 52 or more carbons, the *D_C_* increased between the control and HFD groups, indicating an increase in the concentration of TAG with higher levels of desaturation.

**Table 3:** Weighted saturation index (*D_C_*) of triacylglycerols (TAG) in tumors of Lewis lung carcinoma from mice fed the AIN93G or the high-fat diet.

	nmol/mg _tissue_			*D_C_*	
*C*=	AIN93G	High-fat	Change^a^ %	AIN93G^b^	High-fat^b^
46	0.21	0.10	50	1.08 ± 0.05	1.03 ± 0.03
48	0.97	0.44	45	1.67 ± 0.05	1.70 ± 0.03
49	0.09	0.08	80	–^c^	–
50	3.86	2.95	76	2.39 ± 0.02	2.42 ± 0.03
51	0.26	0.23	91	–	–
52	8.20	13.59	166	3.31 ± 0.02	3.46 ± 0.03
53	0.19	0.30	159	–	–
54	6.94	21.50	310	4.42 ± 0.02	4.80 ± 0.03
56	0.91	1.75	193	5.34 ± 0.08	5.48 ± 0.09
58	0.42	0.78	186	4.01 ± 0.08	4.55 ± 0.21
Total	22.04	41.73	189		

*^a^Values are TAG concentration in high-fat diet-fed mice divided by TAG concentration in AIN93G-fed mice expressed as a percent*.

*^b^Values are mean ± SD (*n* = 6 per group)*.

*^c^Insufficient data for computation*.

While the *D_c_* for the TAG48 and TAG48 clusters do not change between the two groups, they do decrease in concentration. By total TAG concentration for each group, the tumors from HFD-fed mice contained less than 50% of C46 and C48 when compared to tumors from the controls, but had an increase of 159 and 310% of species containing 52 or more carbons thereby contributing to the overall increase in the TAG concentration (Table 3).

**Figure 8:**
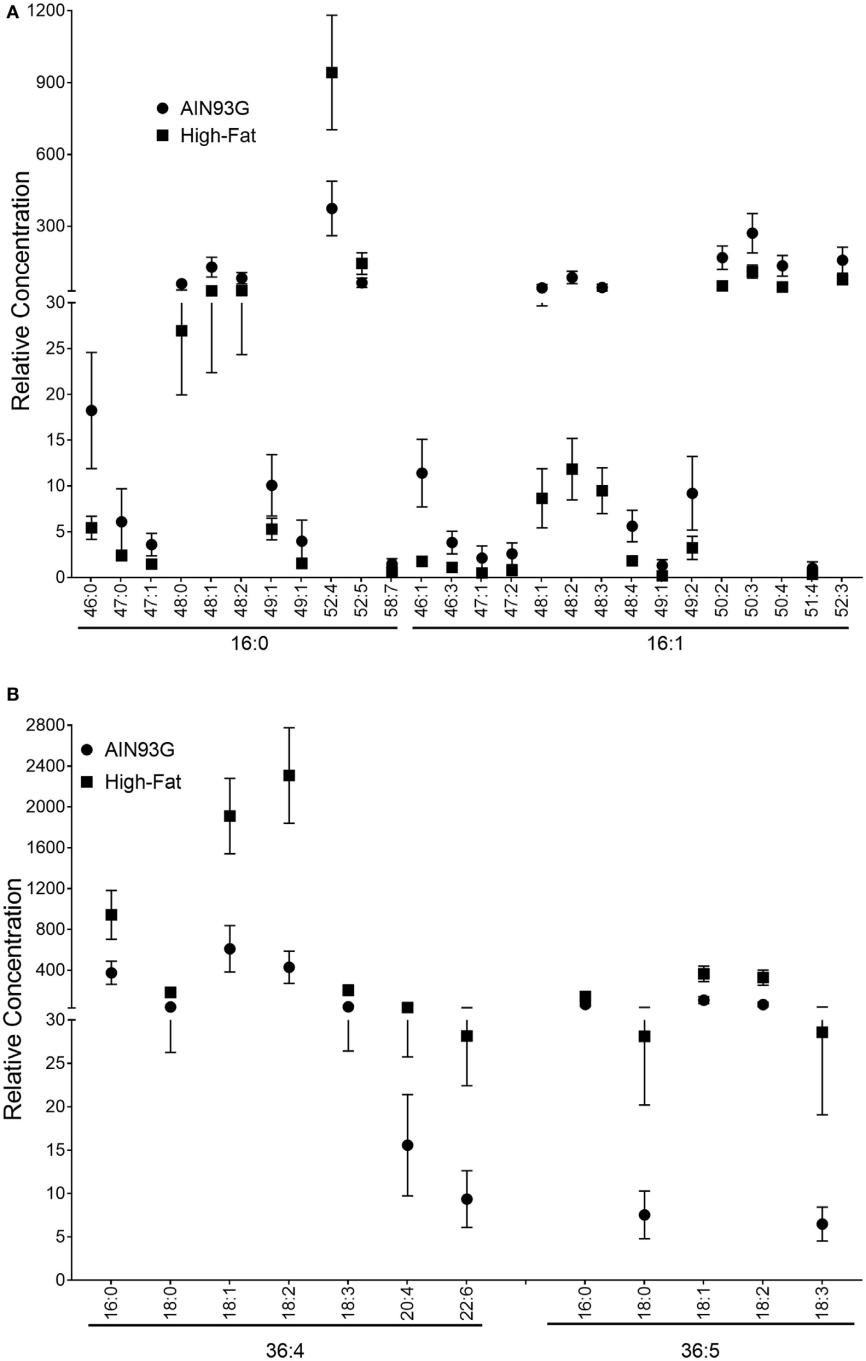
Relative concentrations of triacylglycerols (TAG) corresponding to the fatty acids that contribute to significant (*p* ≤ 0.05) concentration changes including16:0 and 16:1 **(A)** and subtracted from brutto structure including 36:4 and 36:5 **(B)** in the primary tumor of Lewis lung carcinoma from mice fed the AIN93G diet or the high-fat diet (HFD). Values are mean ± SD (*n* = 5 for the AIN93G group and *n* = 6 for the HFD group).

Using neutral loss experiments, it was possible to interpret the differences in TAG concentration between the two groups in terms of specific fatty acid moieties. Decreases in the concentration for TAG48 species corresponded to statistically significant decreases in the same species as observed in neutral loss experiments for both palmitic (16:0) or palmitoleic (16:1) acid (Figure 8A). The only 16:0-containing species to experience significant increases in concentration were TAG52:4 and TAG52:5. When the 16:0 contributions are subtracted from these brutto structures, the remaining acyl chain C and saturation values are 36:4 and 36:5, which correspond to fatty acid combinations of 18:2_18:2 and 18:2_18:3, respectively (Figure 8B). In neutral loss experiments for 18:1, 18:2, 18:3, 20:4, and 22:6, species with the 18:2_18:2 and 18:2_18:3 remainder motifs experienced statistically significant increases in the HFD-fed mice. Values and statistical comparisons for the TAG concentration (for NLS determinations) are provided in Table S5 in Supplementary Material. The increase in these linoleic and linolenic acid-containing species is a reflection of the increased concentration of these fatty acids in the HFD.

### *De Novo* Lipogenesis

**Figure 9:**
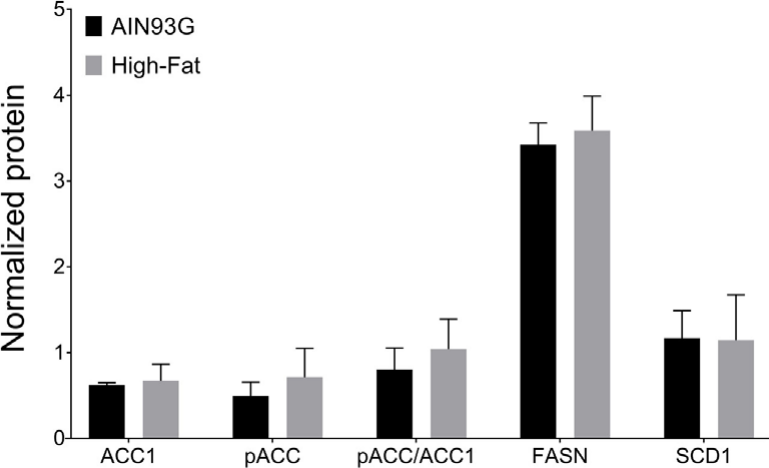
Expression of *de novo* lipogenesis enzymes fatty acid synthase (FASN), acetyl-CoA carboxylase-1 (ACC1), phosphorylated ACC1 (pACC), stearoyl-CoA desaturase-1 (SCD1), and the ratio of pACC to ACC1. The chemiluminescent signals of the target proteins were normalized to the signals of the system control protein. Values are mean ± SD (*n* = 6 per group).

Available studies indicate that the *de novo* lipogenesis pathway proteins are involved with HFD-induced tumorigenicity (40). However, analysis of the de novo lipogenic proteins demonstrated no differences in expression levels of *de novo* lipogenic enzymes ACC1, FASN, and SCD1, nor the pACC/ACC1 ratio, between the two groups (Figure 9).

## Discussion

The alteration of lipid metabolism in cancer cells may affect cancer progression, as evidenced by results demonstrating that lipid profiles of cancer tissues are different from those of adjacent normal tissues (13–15). The composition and concentrations of fatty acids of a diet may alter lipid profiles of cancer, and thus determine the fate of the diet on tumorigenesis (11). LLC is an aggressive carcinoma, which metastasizes spontaneously to the lungs from a subcutaneously transplanted primary tumor (28, 41); feeding mice an obesogenic, HFD enhances its pulmonary metastasis (7, 41). In the present study, consumption of the HFD resulted in quantitative changes in PLs and TAG in the primary tumor of LLC.

Significant alterations in cellular glycerophospholipids occurred in the tumors following intake of HFD. Malignant progression alters the cellular composition of glycerophospholipids and may result in enhanced malignancy as a result of changes in cellular structure and signaling. PC and PE lipids in cancer tissue are altered compared to normal tissue as a result of malignant progression in humans (lung cancer and breast cancer) (17, 18), in rodent models of cancer (P53 and Brca1 double-knockout), and in *in vitro* works (21MT-2 metastatic breast cancer cells vs 76N normal epithelial cells; hormone-resistant MB435 and MB231 breast cancer cells vs hormone-sensitive MCF-7 breast cancer cells) (42, 43).

Our results demonstrate that intake of HFD decreased the content of MUFA and saturated fatty acid (SFA) containing PC and PE lipids in tumors relative to those from normal fat, lean controls; where as PUFA-containing PE and PC lipids were elevated (see Figures 2 and 5). Soybean oil, rich in the n-6 and n-3 PUFAs α-linolenic acid (ALA; 18:3n-3) and linoleic acid (LA; 18:2n-6) respectively, was used in this study as the source of dietary fat as part of the AIN93 formulation (27). Thus, the total amount of fatty acids, but not the proportions of the fatty acids, differed between the dietary groups. The shift in PE and PC composition to a higher PUFA composition in tumors from HFD fed mice is reflective of higher total PUFA intake. The decrease in SFA and MUFA containing PE and PC lipids may be the result of (1) greater selectivity for PUFA vs SFA and MUFA in these PLs, (2) selective shunting of MUFA and SFA to other lipid pools, and/or (3) a decrease in *de novo* synthesis of SFA and MUFA either in tumors or in the mouse.

The infusion MS approach we used is able to determine *sn*1 vs *sn*2 position of fatty acids in PC esters. For most PC esters, the percentage of fatty acid in sn1 vs sn2 is relative stable. For example, PC18:0/18:2 is observed a 90% of the total PC18:0_18:2 species, a percentage that is not altered by intake of the HFD (Table S1 in Supplementary Material). On the other hand, the sn1 regioisomer percentage decreased for palmitic acid-containing PC(16:0/18:3), PC(16:0/18:1), and PC(16:0/18:2). Except for PC(16:0/18:1), the decrease in the sn1 percentage was due to an increase in the content of corresponding *sn*2 regioisomer. The biochemical impact of these changes is not clear. However, such changes in PC structures may impact membrane fluidity or interactions with proteins.

Cancer is metabolically active to provide energy for its rapid development and growth. TAG stored in lipid droplets of the cells, provide a reservoir of fatty acids that can be utilized for energy generation. TAG species are elevated in non-small cell lung cancer compared to the normal lung tissue (14). In this study, the HFD altered TAG composition and concentration in the tumor, evidenced by an increase in the overall concentrations of TAG in the tumor. These findings indicate that consumption of a diet high in PUFAs leads to the incorporation of dietary fatty acids into the TAG species and suggest that exogenous lipids may alter the energy metabolism of LLC cells. Furthermore, the fatty acid reservoir has been suggested to not only serve as an energy depot, but may also have active roles in cancer pathogenesis; cancer promoting cyclooxygenase-2 and prostaglandin synthase are found in lipid droplets of colon cancer cells (23). It remains to be investigated whether the elevation of TAG is purely an increase in energy storage, our findings suggest that this elevation may contribute to the enhanced aggressiveness of LLC.

Several reports indicate that CER metabolism is implicated in tumor development in part through extracellular matrix interactions (44, 45). However, we observed no differences in the concentrations of several CER or HexCer species in tumors as a result of intake of the HFD. While we recognize that this lack of change may be the result of the small number of tumors analyzed, the CER and HexCer concentrations were similar between both groups as opposed to the changes for PC, PE, and TAG. We cannot exclude that this lack of change results from the intake of a high PUFA, obesogenic diet vs a high SFA, obesogenic diet. Intake of a high SFA diet is associated with increases in CER (46, 47).

Several studies describe the ability of tumors to become more lipogenic, thereby giving them the ability to provide their own fatty acids essential for promoting tumorigenesis (40). The expression of *de novo* lipogenic enzymes in this study was not altered by the diets; it suggests that generation of fatty acids through lipogenesis is not likely responsible for the modified fatty acid levels observed. It has been shown that a HFD reduces hepatic *de novo* lipogenesis (48). It is within reason that the HFD may cause liver to reduce production of MUFA and SFA and that the tumor fatty acid profile may reflect hepatic production of MUFA and SFA. We cannot rule out that decreases of MUFA and SFA in TAG may be the result of lipolytic metabolism of MUFA and SFA containing TAG or perhaps modifications in fatty acid transport in the tumor (40, 49). Subsequent tracer-based studies are needed to define mechanisms underlying changes in lipid metabolic pathways.

Our work characterizes the lipidomic profile of LLC in control and obese mice and demonstrated that an obesogenic, HFD altered the lipidome. This alteration reflected the fatty acid composition of soybean oil used in the diet. We demonstrated previously that intake of a HFD enhances lung metastasis from a subcutaneous primary tumor of LLC (8, 50), indicating that HFD increases the aggressiveness of malignant cells. The changes in lipidomic profiles of LLC by the HFD suggest that this alteration may contribute, at least partly, to the enhanced metastasis. Lipidomic research provides us a useful tool in identifying and quantifying thousands of cellular lipid species and their interactions with other nutrients and metabolites. Further studies are warranted to define the biochemical mechanisms of obesity in cancer promotion, so as to the strategies for its prevention, through identifying alterations in cellular lipid metabolism, trafficking, and homeostasis.

## Ethics Statement

This study was performed in accordance with the Guide for the Care and Use of Laboratory Animals of the National Institutes of Health (51) and was approved by the Institutional Animal Care and Use Committee of Grand Forks Human Nutrition Research Center.

## Author Contributions

MP, LY, and SS contributed to the conception and design of the study, data interpretation, and writing of the manuscript. PŽ and MB performed lipidomic analyses. AM performed *de novo* lipogenesis analyses, and they contributed to data interpretation and manuscript preparation. All authors contributed to review and revision of the manuscript and agreed to be accountable for the content of the work.

## Conflict of Interest Statement

The authors declare that the research was conducted in the absence of any commercial or financial relationships that could be construed as a potential conflict of interest.

## Abbreviations

ACC1, acetyl-CoA carboxylase-1; BHT, butylated hydroxytoluene; CER, ceramides; FASN, fatty acid synthase; FDR, false discovery rate; HexCer, hexosylceramide; HFD, high-fat diet; LLC, Lewis lung carcinoma; LPC, lysophosphatidylcholine; LPE, lysophosphatidylethanolamine; MS, mass spectrometry; MUFA, monounsaturated fatty acid; NLS, neutral loss scan; pACC, phosphorylated ACC1; PC, phosphatidylcholine; PE, phosphatidylethanolamine; PIS, precursor ion scan; PL: phospholipids; PUFA, polyunsaturated fatty acid; SCD1, stearoyl-CoA desaturase-1; SFA, saturated fatty acid; sPLS-DA, sparse partial least squares-discriminant analysis; TAG, triacylglycerol.
